# Left atrial appendage thrombus secondary to left atrial ischaemia owing to impaired left atrial branch perfusion

**DOI:** 10.1093/ehjcr/ytac355

**Published:** 2022-08-25

**Authors:** Hironori Ishiguchi, Takayuki Okamura, Masafumi Yano

**Affiliations:** Division of Cardiology, Department of Medicine and Clinical Science, Yamaguchi University Graduate School of Medicine, Ube, Japan; Division of Cardiology, Department of Medicine and Clinical Science, Yamaguchi University Graduate School of Medicine, Ube, Japan; Division of Cardiology, Department of Medicine and Clinical Science, Yamaguchi University Graduate School of Medicine, Ube, Japan

**Figure:**
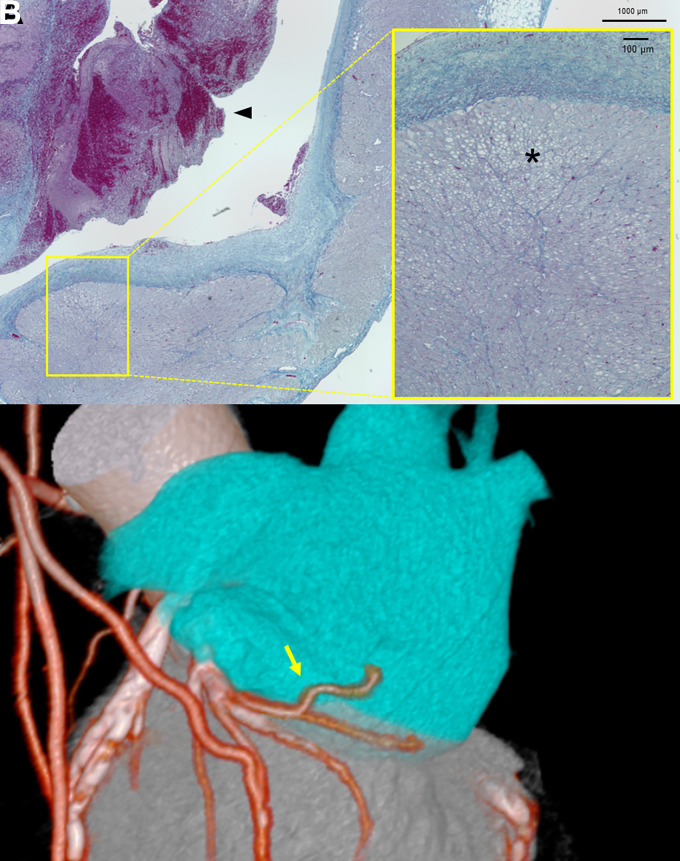


A 64-year-old man without history of atrial fibrillation was admitted for congestive heart failure owing to systolic impairment of the left ventricular posterior–inferior wall (left ventricular ejection fraction, 35%; *Video 1*). He developed chest discomfort several weeks prior, suggesting the aetiology would be a recent myocardial infarction. On Day 3 of hospitalization, he developed acute chest pain with ST-elevation in precordial leads on electrocardiogram (see Supplementary material online, *Figure A*). Coronary angiography revealed occlusion of the middle-left anterior descending (LAD). Moreover, occlusions of the right coronary and left circumflex arteries were also identified (see Supplementary material online, *Figures B–D*, *Video 2*). Coronary slow-flow phenomenon was observed in the left atrial (LA) branch (see Supplementary material online, *Figure D*, arrow). Thereafter primary revascularization of the LAD was accomplished, and emergency coronary artery bypass grafting (CABG) was planned. Pre-surgical contrast-enhanced computed tomography (CT) incidentally detected a thrombus in the left atrial appendage (LAA) (see Supplementary material online, *Figure E*). Transesophageal echocardiography showed impaired LAA contractility (see Supplementary material online, *Figure F*, left: thrombus, right: LAA emptying velocity; *Video 3*). Left atrial appendectomy was performed concomitantly with CABG (see Supplementary material online, *Figure G*). Histological analysis revealed colliquative myocytolysis in the subendocardium of the LAA, indicating acute ischaemia (*Panel A*: low-power field of Azan stain, arrowhead: thrombus, *yellow square*: high-power field, asterisks: area of colliquative myocytolysis). Post-operative CT confirmed the recovery of LA branch flow (*Panel B*: arrow, LA branch). Our case demonstrates that LA ischaemia owing to impaired LA branch perfusion could be a rare mechanism for thrombus formation during normal cardiac rhythm.

## Supplementary Material

ytac355_Supplementary_Data

