# Supplementary figures and images for: Left atrial appendage thrombus secondary to left atrial ischaemia owing to impaired left atrial branch perfusion
Source: Eur Heart J Case Rep. 2022 Aug 25;6(9):ytac355. doi: 10.1093/ehjcr/ytac355 (PMC9536287; doi:10.1093/ehjcr/ytac355)

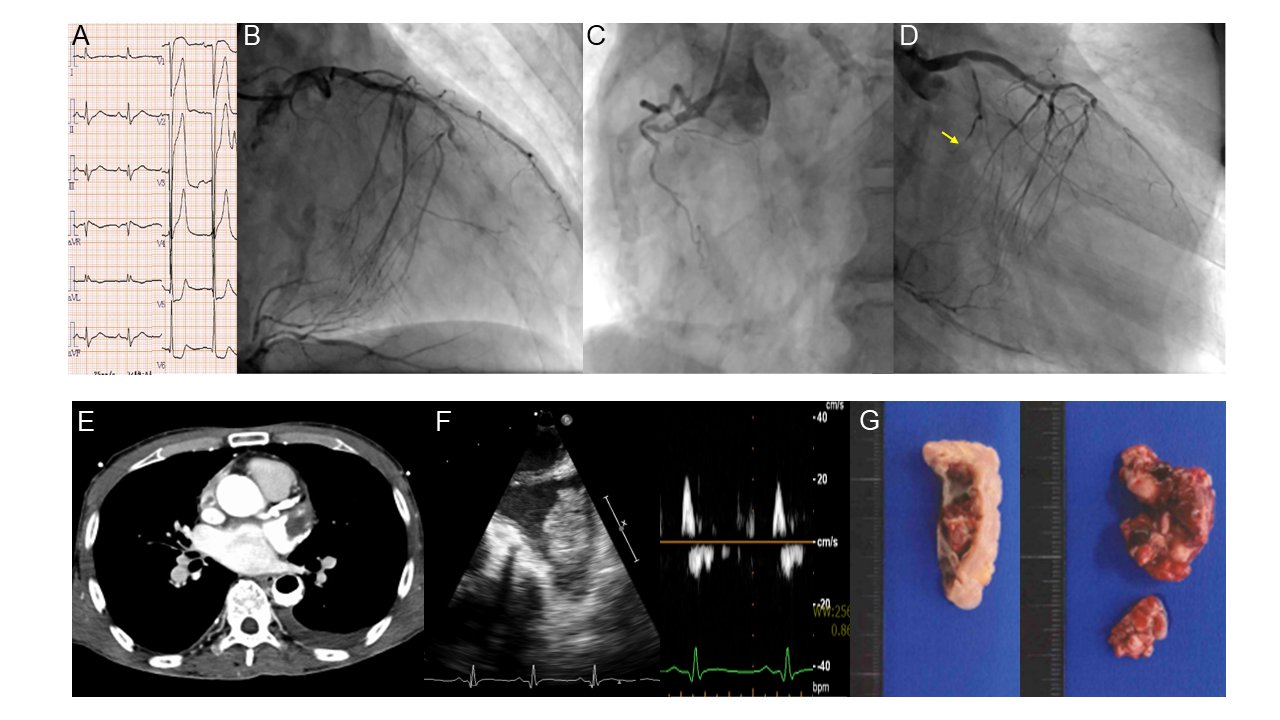

Supplement: ytac355_Supplementary_Data [file ytac355_Supplementary_Data.tif]
